# Surface-Grafted Poly(ionic liquid) that Lubricates in Both Non-polar and Polar Solvents

**DOI:** 10.1021/acsmacrolett.1c00174

**Published:** 2021-06-28

**Authors:** David Burgess, Na Li, Nicole Rosik, Peter J. Fryer, Ian McRobbie, Haining Zhang, Zhenyu J. Zhang

**Affiliations:** † School of Chemical Engineering, University of Birmingham, Edgbaston, Birmingham, B15 2TT, United Kingdom; ‡ State Key Laboratory of Advanced Technology for Materials Synthesis and Processing, Wuhan University of Technology, No. 122 Luoshi Road, Wuhan, 430070, People’s Republic of China; § Innospec Inc., Innospec Manufacturing Park, Oil Sites Road, Ellesmere Port, Cheshire, CH65 4EY, United Kingdom

## Abstract

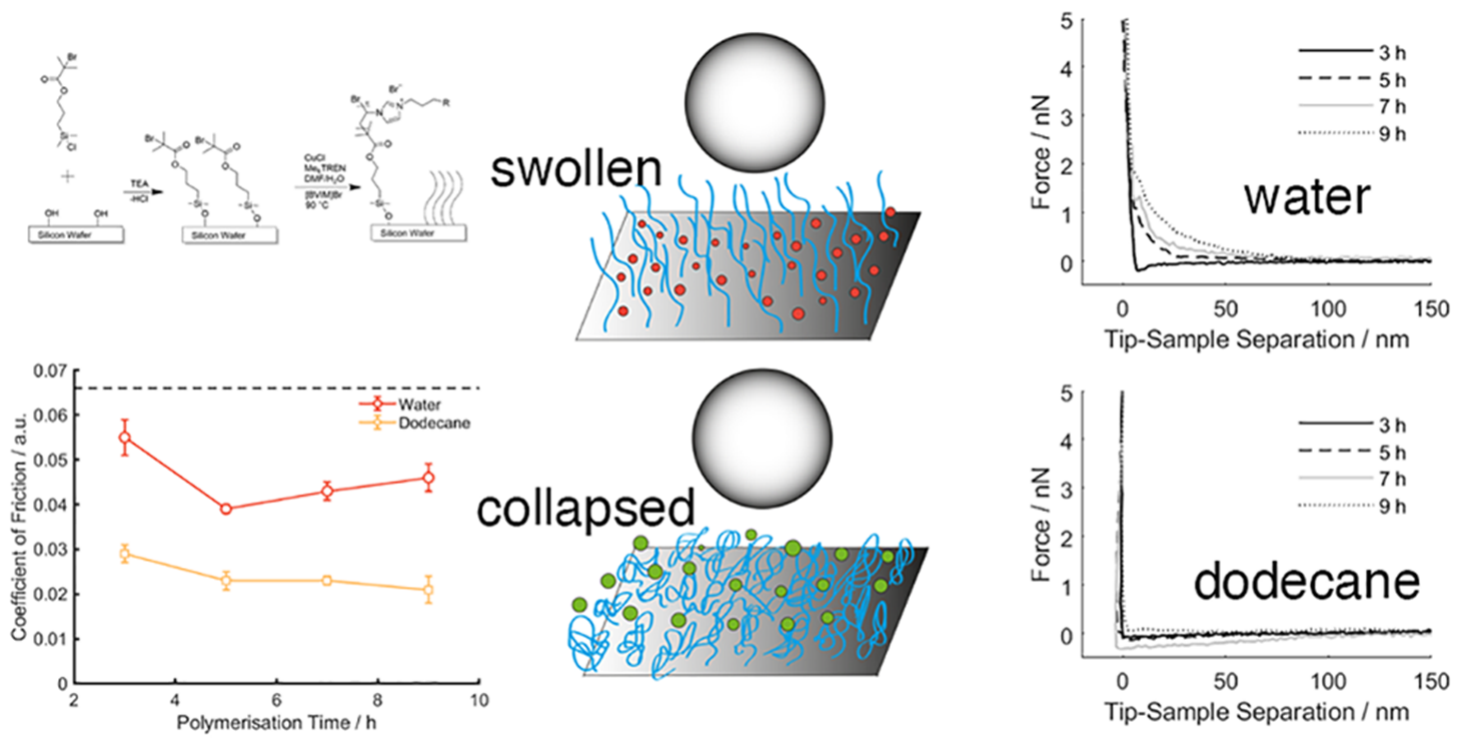

We show that a surface-grafted polymer brush, 1-*n*-butyl-3-vinyl imidazolium bromide-based poly(ionic liquids), is able to reduce the interfacial friction by up to 66% and 42% in dodecane and water, respectively. AFM-based force spectroscopy reveals that the polymer brush adopts distinctively different interfacial conformations: swollen in water but collapsed in dodecane. Minimal surface adhesion was observed with both polymer conformations, which can be attributed to steric repulsion as the result of a swollen conformation in water or surface solvation when the hydrophobic fraction of the polymer was exposed to the dodecane. The work brings additional insight on the polymer lubrication mechanism, which expands the possible design of the polymer architecture for interfacial lubrication and modification.

Surface-grafted polymers are a commonly implemented strategy to render surface properties in effectively adjusting surface interactions by steric repulsion (nonionic polymer) and additional electrostatic interaction (polyelectrolyte).^1−7^ Such a system has demonstrated a great ability to introduce exceptional lubrication capability in both biological and synthetic polymers that were immersed in a range of media. It is explained that the effective reduction in the sliding friction between two surfaces is due to a synergistic effect of resistance to penetration of the one surface into the polymers at the other contacting surface and the fluidity of these polymer brushes, which is a characteristic gained from the solvation layers surrounding the polymer brushes.^8−11^ For example, poly[2-(methacryloyloxy)ethyl phosphorylcholine] showed an unparalleled lubrication performance, with a Coefficient of Friction (CoF, μ) as low as 0.00004 at pressures as high as 7.5 MPa, which is attributed primarily to the strong hydration of the phosphorylcholine-like monomer units known to bind around 15–25 or more water molecules.^12^ The same principle was demonstrated in a nonaqueous environment whereby poly(alkyl methacrylates) brushes were examined in hexadecane.^13^ Polymer brushes tend to demonstrate an exceptional lubrication in one type of solvent, either polar or non-polar.^14−16^ The effect of preferential solvent intake on polymer lubrication was shown by Mathis and colleagues,^17^ whereby poly(dodecyl methacrylate) was exposed to ethanol and an ethanol/toluene mixture at different temperatures. It was reported that the more solvent uptake from the polymer on the surface, the lower the Coefficient of Friction,^17^ thus, demonstrating the importance of the swollen brush configuration in reducing friction.

Using ionic liquids as lubricant additive is another strategy to reduce interfacial friction.^18,19^ Alkyl-imidazolium tetrafluoroborate ionic liquid (IL) was one of the first to show excellent friction reduction properties between steel–steel contacts.^20^ It was proposed that IL molecules could form alternating layers of cations and anions at the articulating interface, keeping the surfaces in contact separated at the molecular scale and subsequently reduce interfacial friction.^21^ These layers slide over one another with the absence of a stick–slip (thereby preventing wear also) and potentially melt under shear at the yield point, reducing the friction synergistically.^21^ In a recent work, other types of ILs showed good friction reducing properties; most of these contain imidazolium moieties and often tetrafluoroborate or hexafluorophosphate moieties.^22^ As lubricant additives, ILs present potential in greatly reducing the Coefficient of Friction in water- and oil-based solvents, even at low concentrations:^23−29^ the surface adsorbed multilayer film could reduce the interfacial friction and wear.^30,31^

To combine the advantages offered by both surface grafted polymer brush and ionic liquid, poly(ionic liquid)s (PILs) have been developed as a novel platform for a wide range of applications such as thermoresponsive materials, CO_2_ capture and separation, and biosensors.^32^ A series of 1-*n*-butyl-3-vinyl imidazolium bromide ([BVIM]Br)-based PIL, shown in Figure 1, were grafted from silicon substrate under controlled polymerization times (each with a CH_3_ R group), with different functional groups (each with a polymerization time of 5 h) attached to the polymeric backbone, as detailed in our previous publications.^33,34^ Their ability in modulating surface interactions via changes in their interfacial conformation, when being exposed to water or dodecane, was investigated systematically in the present work.

**Figure 1:**
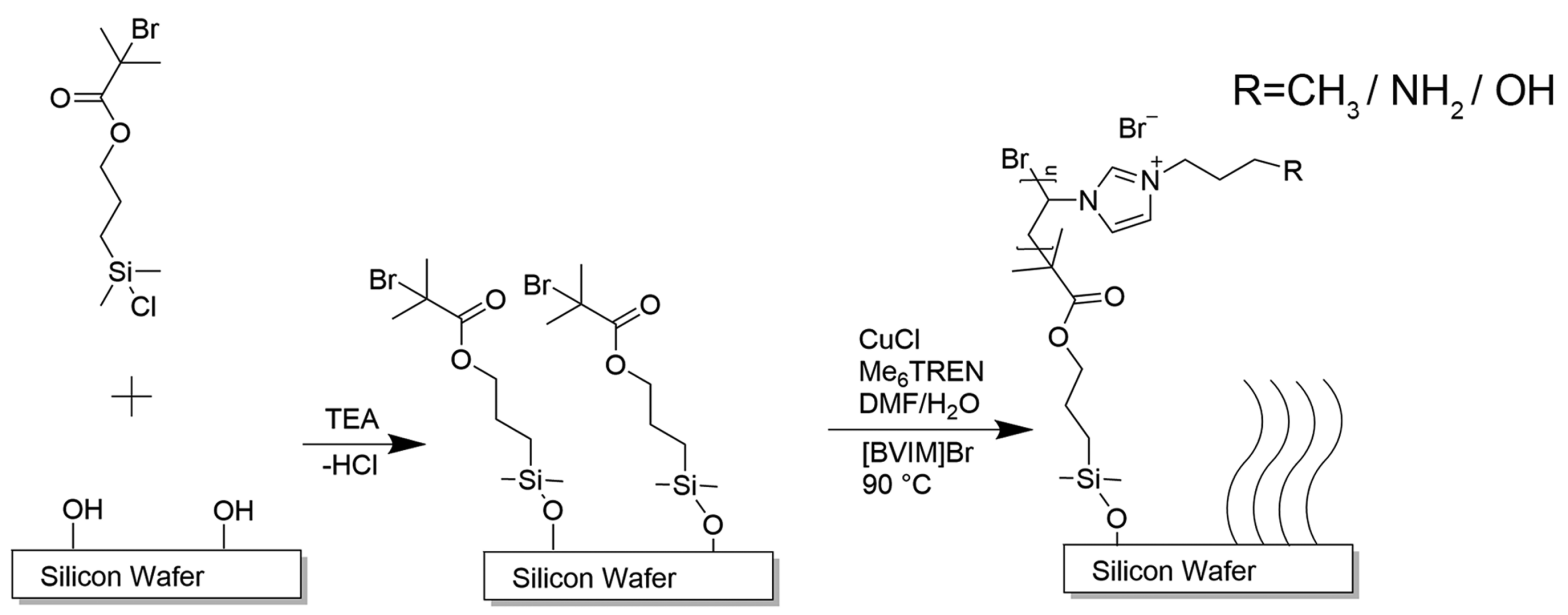
Schematic diagram of the synthesis process of the surface-grafted PIL brushes.

Surface morphology of the PIL samples was acquired in ambient (21 ± 3 °C, RH 20–25%), water, and dodecane, using atomic force microscopy (AFM). Three different scan sizes, 1 μm × 1 μm, 5 μm × 5 μm, and 10 μm × 10 μm, were captured from five different locations randomly on each PIL sample to ensure the images and data are representative and statistically reliable. Images (1 μm × 1 μm size) of the PIL sample with 5 h polymerization time are presented in Figure 2 to highlight the surface morphology and the effect of solvent on polymer conformation. A homogeneous surface characteristic was observed across all PIL samples of different polymerization times and different R groups, confirming the presence of the PIL brush, comparing to cleaned silicon wafers that are atomically flat.^35^ Uniform layers were observed in both solvents (Figure 2b,c). Surface roughness (*R*_a_) of the PIL films is 1.0 ± 0.2, 1.0 ± 0.3, and 3.0 ± 0.5 nm in ambient, dodecane, and water, respectively. The *R*_a_ values were found to be very similar in ambient and dodecane, which implies that the surface-anchored polymer chains had a similar conformation, while that in water was nearly trebled, suggesting that the PIL brushes were well solvated. Thickness of the polymer brush layer in ambient is between 6.5 and 11.2 nm, as characterized by ellipsometry (details are presented in the Supporting Information).

**Figure 2:**
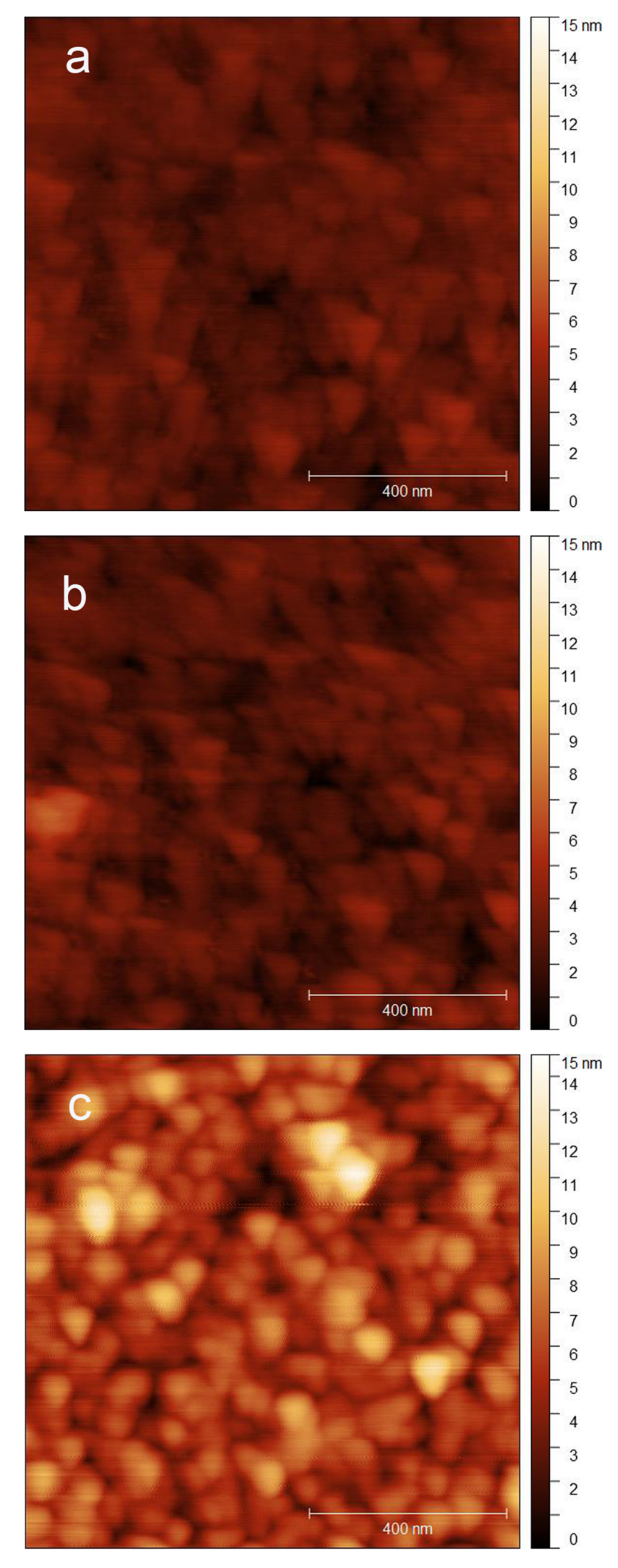
Surface morphology images of the PIL brushes containing a CH_3_ group (polymerization time of 5 h) in (a) ambient, (b) dodecane, and (c) water.

To evaluate the polymer–solvent interaction, contact angles of water and diiodomethane were measured on the PIL samples (Figure 3a) to calculate the polar energy component, dispersive energy component, and the total surface free energy using the harmonic mean method.^36,37^ The water contact angles are in agreement with those acquired on poly(1-(4-vinylbenzyl)-3-butyl imidazolium hexafluorophosphate) (PVBIm-PF6) grafted from a planar silicon substrate, confirming the rather hydrophobic nature of the PIL film.^38^ There was no difference in surface energy between the samples of different polymerization times (see Supporting Information), which is not surprising, as the chemical nature and grafting density were similar. However, the surface energies of the PIL samples synthesized with varying functional groups differ distinctively, showing the impact of the R group on the preference toward solvents. Figure 3b shows surface free energies as a function of the R groups: the polar component of the surface free energy shows little difference between the PIL samples, implying a similar magnitude of preference toward water. However, the dispersive component of the PIL samples varies notably, which contributes to the overall differences in surface free energy: the PIL brush containing NH_2_ group has the lowest dispersive component, followed by PIL-CH_3_, with PIL-OH having the greatest dispersive component. It is worth noting that the dispersive component appears to be much greater than the polar components for all PIL samples, highlighting the PIL brush prefers non-polar solvent to polar solvent. Based on the AFM morphological images (Figure 2), it is probable that the grafted PIL molecules are extended from the supporting substrate once immersed in a good solvent (water in the present study) and, consequently, expose the polar groups to the solvent.^39^ However, the PIL brush would adopt a collapsed conformation in dodecane, and its degree of solvation would be determined by the available imidazolium and R groups.

**Figure 3:**
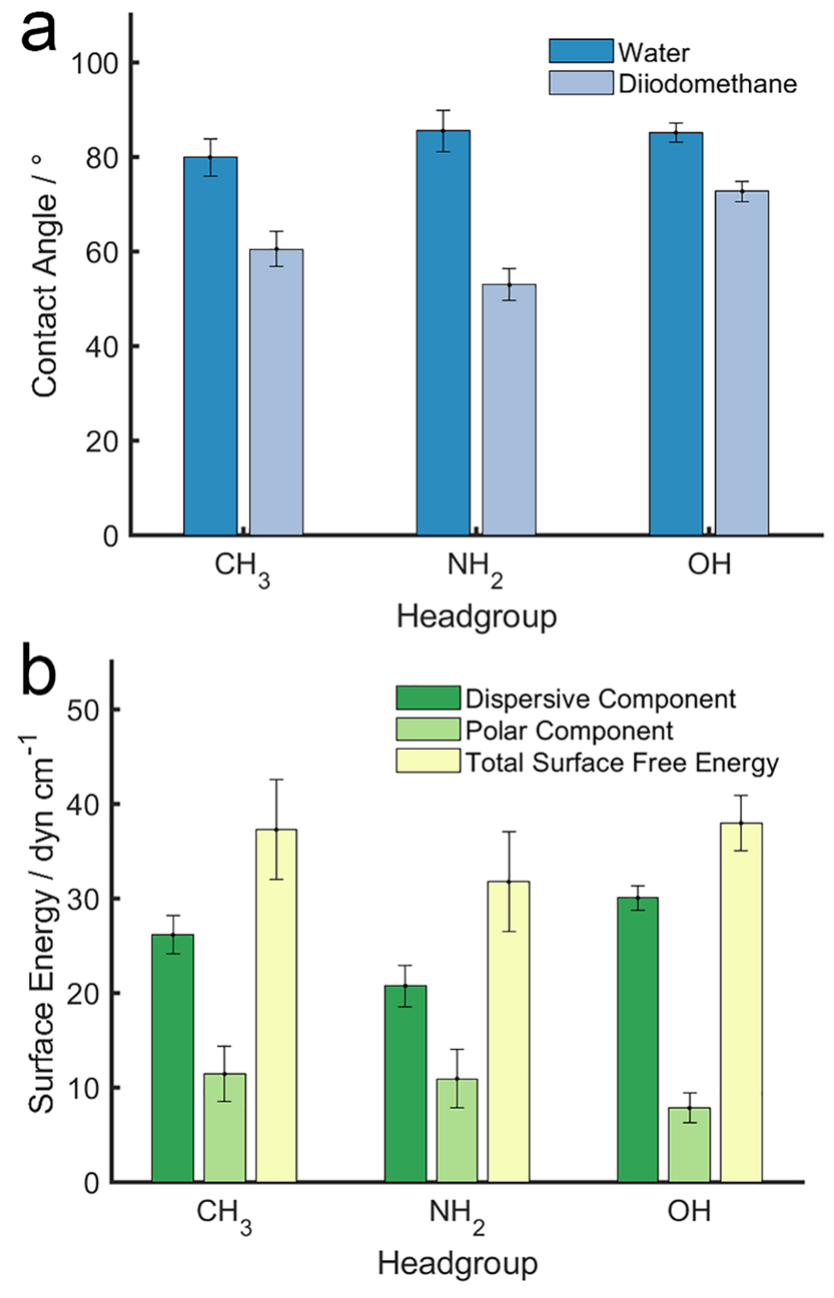
(a) Contact angles of water and diiodomethane on the PIL samples that were polymerized for 5 h; (b) Corresponding surface energy of the PIL samples.

The tribological characteristics of the PIL brushes were evaluated by measuring the Coefficient of Friction using a reciprocating ball-on-plate tribometer with a sliding velocity of 0.5 mm s^–1^. A Hertzian contact pressure of approximately 381.3 MPa was produced using a 2 mm borosilicate glass sphere with a normal load of 100 g (equivalent to 0.98 N), assuming Poisson’s ratios and Elastic Moduli are 0.2 and 72 GPa for glass^40^ and 0.17 and 70 GPa for silicon wafers,^41^ respectively. It is worth noting that the actual contact pressure could be slightly less due to an increased contact area when the PIL brush is in a swollen state. The applied load was chosen in accordance to a series of macroscopic tribological studies whereby surface grafted polymer brushes exposed to high contact pressure.^13,42,43^ Although a full Stribeck curve was not established in the present work, previous study^42^ suggests that the high applied load (0.98 N), low sliding velocity (0.5 mm s^–1^), and low kinematic viscosity of water (1 cSt) would likely fall into the boundary lubrication regime where two surfaces are in close contact. Figure 4 presents the Coefficients of Friction as a function of polymerization time and functional groups, in both water and dodecane. For each sample, similar CoF values were obtained when several vertical loads (0.2, 0.49, and 0.98 N) and sliding velocity (0.1 and 1 mm s^–1^) were used (see Supporting Information), which provides further evidence that the contact was in the boundary lubrication regime where friction is independent of sliding speed.

**Figure 4:**
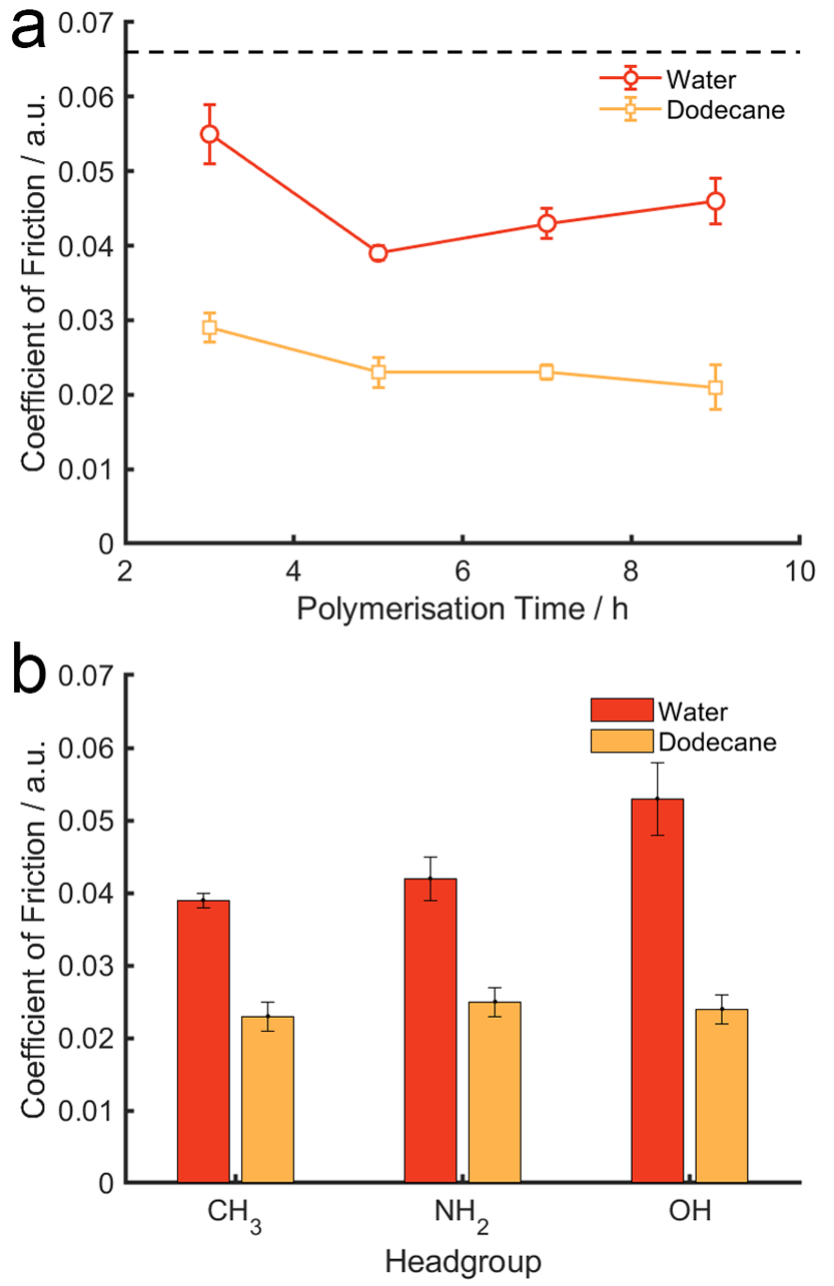
Coefficient of Friction measured on the PIL samples as a function of (a) polymerization time and (b) functional groups under polymerization of 5 h. Error bars represent standard error over five repeats. The Coefficient of Friction on a bare silicon wafer (dotted line in Figure 4a) in both solvents was 0.067.

The CoF on a bare silicon wafer in both solvents was measured to be 0.067 (as shown by the dotted line in Figure 4a), which was reduced with the presence of PIL samples in both solvents, demonstrating that the surface-grafted PIL can lubricate in both aqueous and organic environments. Of the PIL brushes prepared, the maximum CoF (0.055) was acquired on the sample polymerized for 3 h in water, which was reduced to 0.043 on the sample polymerized for 5 h and increased slightly as the polymerization time extended to 7 and 9 h. Unlike that in water, the effect of polymerization time (thickness of the film) on the frictional characteristics of PIL is not notable in dodecane. The incorporated functional groups appear to influence the CoF in water, which is highly likely because the polar groups (NH_2_ and OH) are able to introduce electrostatic interactions with the negatively charged borosilicate sphere in the aqueous medium. However, such effect is diminished in dodecane whereby CoF is independent of the functional groups, which evidences the contribution of electrostatic interaction toward the overall surface forces in the aqueous solution.

Overall, the most striking observation is that the CoF for all PIL samples is nearly reduced by half in dodecane than in water, which is in agreement with the surface energy data that shows the dispersive component is much greater than the polar components for all PIL samples (Figure 3). Adjusting the tribological characteristics of a polymeric system, for example, surface-grafted brush, hydrogel, by changing its molecular configuration upon solvent is well documented, and has been demonstrated multiple times in the previous reports.^45−47^ It is widely accepted that the surface-grafted polymeric chains are well solvated in a good solvent: steric repulsion could introduce lubrication by reducing the surface adhesion. Once being exposed to a poor solvent, surface-grafted polymer chains have less favorable interaction with the solvent molecules, and hence, prefers to interact with the surface in contact, resulting in an increased surface adhesion and friction. It is therefore unusual that the PIL brushes are capable of delivering surface lubrication in both aqueous and organic solvents, as we demonstrate in the present work.

To establish the molecular mechanism that underpins the tribological data (Figure 4), surface adhesion between a borosilicate glass probe (10 μm) and the PIL surfaces in water and dodecane were measured using AFM-based force spectroscopy. The quantified surface adhesion (Figure 5) is in the region up to 9 nN, which is consistent with previous colloidal force spectroscopy studies.^48−50^ The adhesion value in water appears to increase significantly, from 2 to 9 nN, as the polymerization time increases, confirming that there is a growing interaction between the PIL brush and the borosilicate probe. In general, the adhesion force is lower in dodecane than in water, which is consistent with the result of the macroscopic tribological measurements. We postulate that there are two different lubrication mechanisms:

- The PIL brush is well solvated in water, which provides the interfacial lubrication as expected. When separating the two surfaces in contact, there is a strong attractive interaction between the borosilicate probe and the PIL brush, which is controlled by the functional groups incorporated. Being well-solvated, PIL samples with long polymerization times are thicker than those of short polymerization time, which offers a greater contact area with the borosilicate probe, resulting in an increased surface adhesion in water.
- The PIL brush is not solvated in dodecane and, hence, adopts a collapsed conformation. However, the PIL brush has little interaction with the surface in contact (borosilicate probe), as evidenced by the minimal surface adhesion measured, which not only produces a minimal contact area but also a low CoF. It is very probable that the collapsed chains form a compact polymeric layer that is energetically unfavorable to penetrate or perturb, which is uncommon but not impossible. A similar observation was made on a surface-grafted PMPC that did not swell into a brush configuration in 2-propanol.^45^ In such condition, the collapsed interfacial conformation of the polymer resulted in a substantially increased Coefficient of Friction against AFM cantilevers functionalized by COOH and NH_2_ self-assembled monolayers, but a much reduced CoF against a CH_3_ functionalized cantilever.

**Figure 5:**
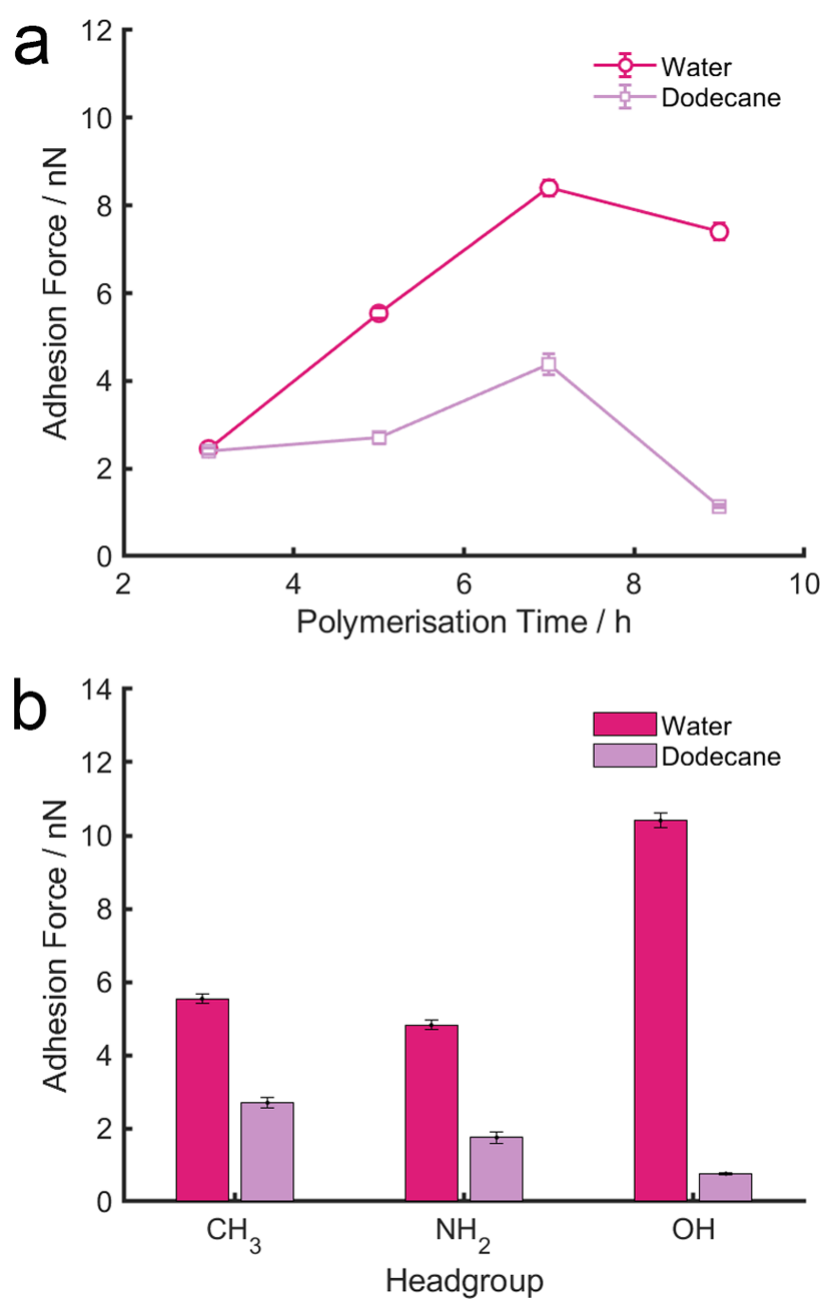
Surface adhesion acquired on the PIL samples as a function of (a) polymerization time and (b) different functional groups using colloidal force spectroscopy.

Results so far support the possibility that the PIL brushes adopt an extended (swollen) conformation when being exposed to water, which induces steric repulsion^2,4^ and subsequently reduces interfacial friction compared to a bare silicon wafer. The increased adhesion observed on the samples with long polymerization time is likely due to the increased contact area. It is striking that much less surface adhesion was measured on the same set of PIL samples in dodecane when the polymer chains adopt a collapsed conformation. It is very likely that the PIL chains form a compact and dense molecular layer that does not interact with the borosilicate probe, which consequently reduces the CoF because of the minimal interfacial interaction.

The surface interactions acquired when the borosilicate colloidal probe was approaching the PIL samples were analyzed to validate the hypothesis that the PIL is solvated to lubricate in water, but is collapsed on the surface to form a compact layer in dodecane with minimal surface interaction. As demonstrated in previous studies, such a method offers both a qualitative judgment on the solvation state of the surface-grafted polymer and a quantitative approach to evaluate the thickness of the brush layer by measuring the onset of repulsion upon contact.^16,45,51−54^Figure 6 shows representative plots of the approaching curves of all the PIL samples acquired in water and dodecane.

**Figure 6:**
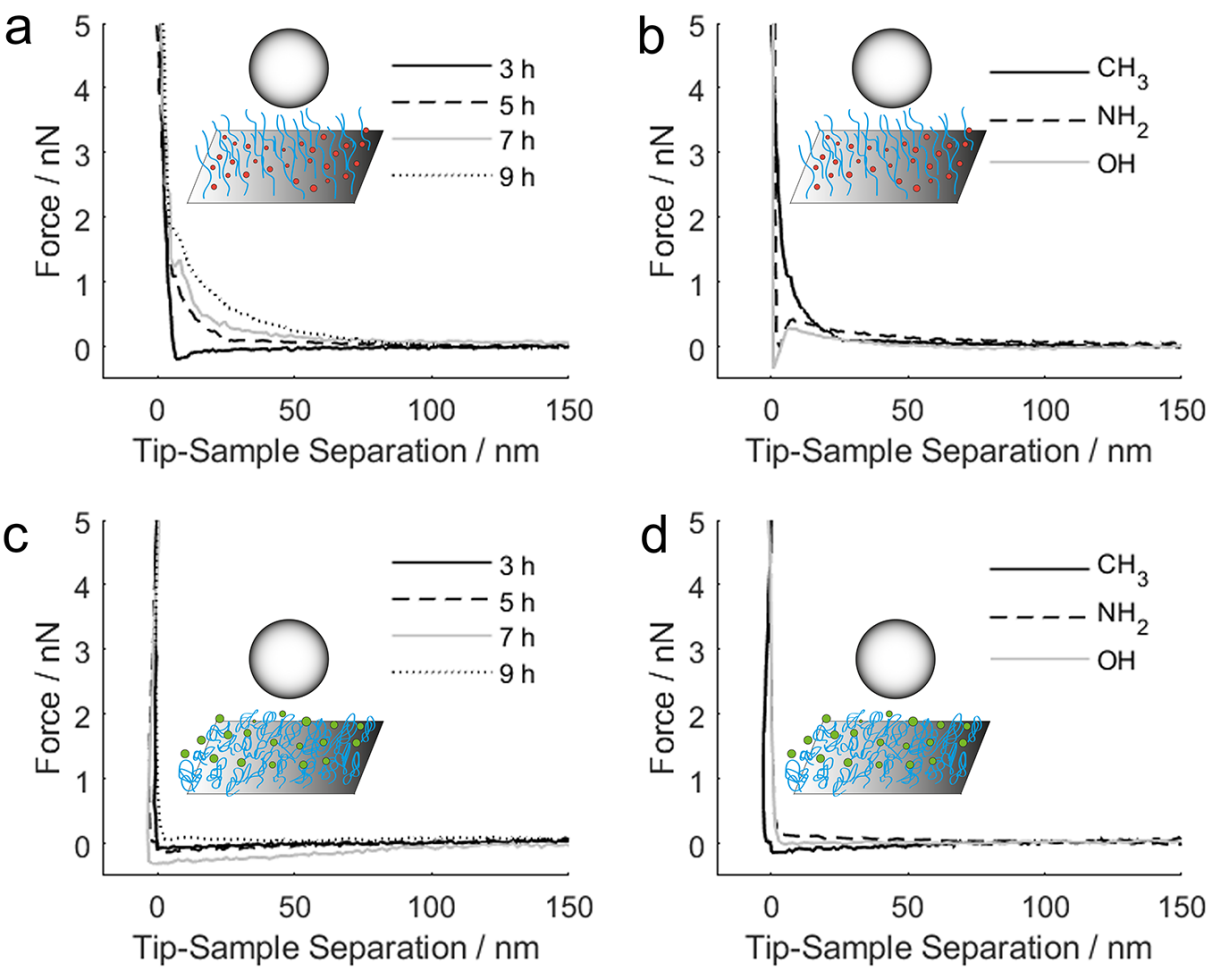
Interfacial interactions measured by the AFM when approaching a borosilicate particle of 10 μm toward the PIL samples in (a, b) water and (c, d) dodecane as a function of polymerization time and functional groups.

In Figure 6a, it can clearly be seen that, as the polymerization time increases, the onset of repulsion begins at a long-range (∼80 nm) and is more pronounced, suggesting that the PIL polymer film was well solvated in water and the chains extended far from the supporting substrate, hence, inducing the steric repulsion. The onset of repulsion correlates well with the polymerization time, except that the one with a 3 h polymerization time shows no onset of repulsion, supporting that the swollen brush configuration is not notable, hence, the high Coefficient of Friction. A distinctively different characteristic was observed once the PIL samples were exposed to dodecane (Figure 6c): the long-range repulsion diminished for all samples. Because there is no electrostatic interaction in non-polar solvent, such as dodecane, the only plausible explanation for the disappearance of such repulsive interaction is the lack of steric repulsion, which confirms that the PIL brushes indeed adopt a collapsed conformation in dodecane. This explanation is consistent with the surface morphology results (Figure 2b,c) that show the overall roughness of the PIL brush surface is greater in water than in dodecane, which is due to its swollen conformation. In Figure 6b it can be seen that there is an exclusive “snap-in” characteristic (ca. 10 nm), an indication of attractive interaction, for the PIL samples containing NH_2_ and OH groups, despite an onset of repulsion around the same distance for all of the PIL samples. This confirms that the attractive interactions are present between the hydrophilic borosilicate probe and the PIL samples containing specific functional groups (−OH and −NH_2_) in water. Such a “snap-in” characteristic of the attractive interaction was no longer observed in Figure 6d, confirming that the attraction is likely electrostatic in nature. This affirms that the lubrication mechanism changes as the surrounding environment is altered from polar to non-polar liquid. The PIL brushes form a swollen and lubricating layer in water, but a compact layer in dodecane that inhibits any attractive interactions with the borosilicate probe.

The tribological characteristics of surface-grafted imidazolium-based poly(ionic liquid)s were quantitatively measured in both water and dodecane. It was found that the PIL brush can deliver an excellent lubrication behavior when it is either swollen or collapsed, as examined by a ball-on-plate tribometer: the Coefficient of Friction reduced by 66% and 42% in dodecane and water, respectively, when being compared with a bare silicon wafer. In addition to the commonly observed polymer lubrication that is introduced by the steric repulsion, we suggest that a collapsed polymer brush can equally offer exceptional surface lubrication, so long as that the surface adhesion with the substrate in contact is minimized. From the tribological perspective, such a collapsed conformation generates the least possible contact area and, hence, could be a potential route in lubricating an articulating interface for a range of possible tribiological applications.

## Supporting Information Available

Materials and methods; Brush thickness; Effect of applied load; Effect of polymerization time on surface energy; Force measurement by polystyrene particle (PDF)
