# Supplementary material for: Surface-Grafted Poly(ionic liquid) that Lubricates in Both Non-polar and Polar Solvents
Source: ACS Macro Lett. 2021 Jun 28;10(7):907–13. doi: 10.1021/acsmacrolett.1c00174 (PMC8296680; doi:10.1021/acsmacrolett.1c00174)
Supplement: Supplementary file 1 — mz1c00174_si_001.pdf [file mz1c00174_si_001.pdf]

SUPPORTING INFORMATION

SURFACE GRAFTED POLY(IONIC LIQUID) THAT LUBRICATES IN BOTH NON-POLAR AND POLAR SOLVENTS

David Burgess,[a] Na Li,[b] Nicole Rosik,[a] Peter J. Fryer,[a] Ian McRobbie,[c] Haining Zhang,[b] and Zhenyu J. Zhang[a*]

a- School of Chemical Engineering, University of Birmingham, Edgbaston, Birmingham, B15 2TT United Kingdom

b- State Key Laboratory of Advanced Technology for Materials Synthesis and Processing, Wuhan University of Technology, No. 122 Luoshi Road, Wuhan, 430070 P.R. China

c- Innospec Inc., Innospec Manufacturing Park, Oil Sites Road, Ellesmere Port, Cheshire, CH65 4EY United Kingdom

*Corresponding author email: Z.J.Zhang@bham.ac.uk

Materials

Silicon wafers (‹100› orientation, boron doped, polished one side) were purchased from LiJing Technology (Zhejiang, China). Tris[2-(dimethylamino)ethyl]amine (Me_6TREN), 1-bromobutane, 1-vinylimidazole, 2-bromoethylamine hydrobromide, 2-bromoethanol, 2-bromo-2-methylpropionic acid bromide, and dimethylchlorosilane were purchased from Aladdin (Shanghai, China) and used as received. Chloroplatinic acid were purchased from Alfa Aesar. Copper(I) chloride and allyl alcohol were purchased from Sinopharm Chemical Reagent (China). Deionised water (18.2 MΩ cm^{-1}) generated by a Ulupure-H ultrapure water generator (Ulup, China) was used for PIL synthesis. Pure water (HPLC grade) and *n*-dodecane (≥99%) used in friction and AFM measurements were purchased from Sigma Aldrich (Dorset, UK).

Silicon wafers were immersed in "piranha" solution (98 wt% concentrated sulfuric acid: hydrogen peroxide = 3:1 (vol%)) for 24 hours and then washed three times with deionized water, absolute ethanol, and acetone, respectively and finally stored under a nitrogen environment. Toluene was distilled over sodium using benzophenone as an indicator and stored with molecular sieves. Triethylamine (TEA) and

dichloromethane were refluxed over CaH_2 for 24 h and subsequently distilled under a nitrogen environment.

Synthesis of PIL brushes

Full details of the synthesis can be found in our previous work.[1] In brief, [BVIM]Br (5.8 g, 25 mmol) in 2 mL deionized water, CuCl (30 mg, 303 μmol), and Me_6TREN (0.2 ml, 750 μmol) in 5.8 mL DMF were added to a Schlenk flask containing 1×1 cm initiator-treated silicon wafer pieces under nitrogen protection. Once being mixed evenly, the reaction system was carefully degassed through three freeze-thaw cycles to remove the dissolved oxygen and heated to 90°C in an oil bath for polymerization. The system was allowed to cool down to room temperature once the desired polymerisation time was reached. Silicon wafers were subsequently transferred to a Soxhlet extractor and rinsed with deionized water for 24h to remove any free polymers and unreactive monomer in solution. Finally, the dried silicon wafers were stored in a nitrogen environment. Synthesis of PIL samples containing amino (NH_2) and hydroxyl (OH) groups followed the same procedure.

Tribology measurement

A home build Longshore microscopic reciprocating ball-on-plate tribometer was used to measure friction on the PIL samples under various conditions.[2-4] A borosilicate glass sphere of 2 mm diameter was used to slide across the surface of the PIL substrate over a distance of 40 mm at a speed of 0.5 mm s^{-1}. The measurement constitutes 20 cycles, from which the averaged Coefficient of Friction was taken.

Atomic force microscopy

An atomic force microscope (Dimension 3100, Veeco, UK) with a Nanoscope 4 controller was used for characterising the PIL samples. Gold coated cantilevers with a nominal spring constant of 5 N m^{-1} (Tap150 GB-G, Budget Sensors, Bulgaria) were used to acquire surface morphology. Adhesion force measurements were carried out using a colloidal probe prepared by gluing a borosilicate glass microsphere (Dukes Standards, Thermo Fisher Scientific, USA) with a diameter of 10 μm onto a tipless silicon nitride cantilever with a nominal spring constant of 0.08 N m^{-1} (PNP-TR-TL, Nanoworld, Switzerland). A two-components epoxy resin was used (Araldite, Switzerland). The prepared colloidal probe was left for 24 hours to ensure the adhesive had fully set before use. Adhesion force measurements were carried out by obtaining

100 force curves in a 1 μm^2 area each curve 100 nm apart in a 10 x 10 grid in both liquids. From the force curves, the adhesion force was taken as the pull off deflection.

Surface Energy measurements.

Surface energy was calculated by measuring the contact angle of water and diiodomethane on the PIL sample surfaces. The contact angle was measured using a contact angle goniometer (Ossila, UK). The surface energy was then calculated from the two average contact angles using the harmonic mean method.[5]

Brush thickness characterisation

An ellipsometer (AlphaSE, J.A. Woollam, USA) was used to measure the thicknesses of the PIL brush layer silicon wafer in ambient. A cleaned silicon wafer without any coating was used to fit substrate optical properties. The dry thickness of each PIL brush layer was fitted using a Cauchy model, with a refractive index given by 1.45 + $0.01/\lambda_2$, where the wavelength, λ, is in micrometers. Ellipsometry measurements were performed over three positions on the same sample in ambient.

As explained in a previous work,[1] measuring thickness of polymer brush in liquid when it is in a swollen state is a challenging task. We therefore determined the thickness of the polymer brush based on the force curves acquired when approaching the PIL samples with the AFM colloidal probe. It is a method commonly used in the past.[6-9] The onset of the repulsive interaction, as observed in the approaching part of the force curve recorded, is attributed to the steric repulsion induced by the polymer brush when being compressed. The onset distance values for all four PIL samples are presented in Table S1, alongside the thickness in ambient, measured by the ellipsometer.

Polymerisation time / h	Thickness / nm	
	Ambient (ellipsometry)	Water (AFM based force spectroscopy)
3	8.6 ± 2.1	15 ± 1
5	8.1 ± 1.1	52 ± 4.5
7	6.5 ± 3.6	67 ± 4.8

9	11.2 ± 4.2	82 ± 3.4

Table S1. Thickness of PIL samples of four different polymerisation time, characterised in ambient and water by ellipsometer and AFM respectively.

Effect of applied load

As defined by the tribological principles, Coefficient of Friction is dependent on the lubrication regime that is controlled by contact pressure, sliding velocity, and viscosity of the liquid environment. Several loading forces were chosen according to a series of macroscopic tribological measurements of surface grafted polymer brush,[10-12] in which a similar contact geometry (glass sphere of several mm diameter against silicon wafer on which polymer brush was grafted) and contact pressure (several hundred MPa) were implemented.

Four applied forces were evaluated (0.2 N, 0.49 N, 0.98 N, and 1.96 N): the highest force (1.96 N) was found to result in a noticeable wear track on the silicon wafer, which is not suitable for studying the lubrication of the polymer brush; whilst the modest and low applied forces did not cause any noticeable wear to the sample surface. More importantly, nearly identical Coefficient of Friction values were acquired from all four samples under 0.98 N and 0.49 N, as shown in the graph below. This confirms that the frictional measurements were carried out under boundary lubrication regime where two surfaces were in close contact.

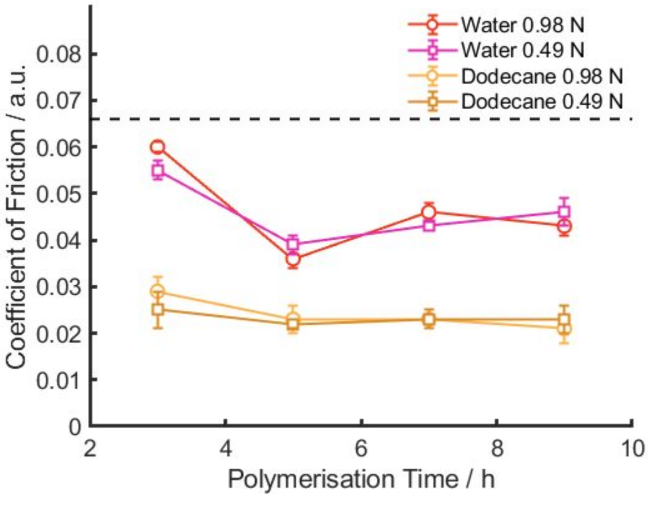

Figure S1. Coefficient of Friction measured on the PIL samples as a function of applied load.

Based on the assumption that the contact mechanics follows Hertzian model, and the information presented in the table below, we estimated that the contact area and contact pressure involved adhesion and friction experiments.

	Friction		Adhesion	
	Glass Sphere	Silicon Substrate	Glass Sphere	Silicon Substrate
Poisson's ratio	0.2 [13]	0.17 [14]	0.2 [13]	0.17 [14]
Elastic modulus	72 [13]	70 [14]	72 [13]	70 [14]
Sphere diameter (m)	0.002		1×10^{-5}	
Contact area (m^2)	2.68×10^{-9}		2.3×10^{-16}	
Applied load (N)	0.98		5×10^{-9}	
Contact pressure (MPa)	381		22	

The sliding speed was chosen to ensure that the measurement was in the boundary lubrication regime whilst balance the data acquisition rate. We found that an increased sliding speed (1 mm s^{-1}) resulted in data with more noise, and the slow sliding speeds (0.1 mm s^{-1}) showed little to no changes in the CoF.

Effect of polymerisation time on surface energy

Surface energy measurements were carried out on the whole range of PIL samples, which is presented in Figure S2 as a function of polymerisation time. As anticipated, there is very little difference in both polar and dispersive components between the PIL samples since their chemistry is identical. This highlights that the polymer chain length does not contribute towards surface energy.

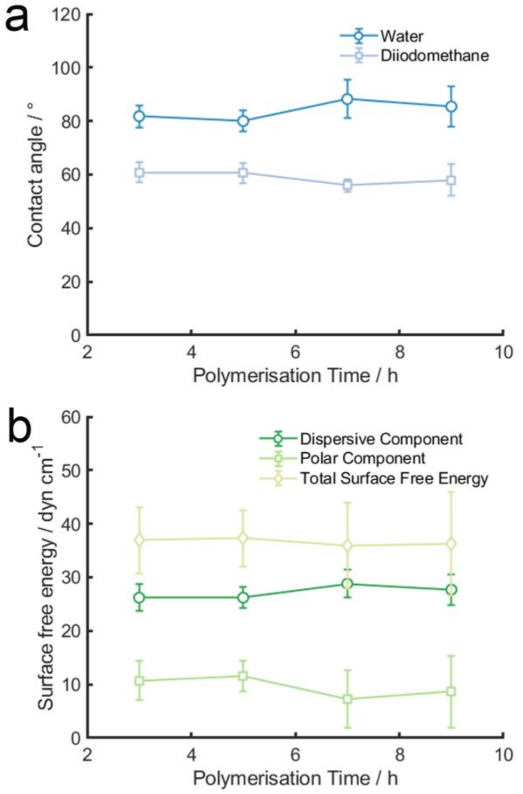

Figure S2. (a) Contact angles of water and diiodomethane on the PIL samples that were polymerised for 3, 5, 7, and 9 hours; (b) Corresponding surface energy of the PIL samples.

Force measurement by polystyrene particle

Identical AFM-based surface interaction measurements were carried out with a neutral polystyrene particle of 20 μm diameter, of which results are shown below. There is very little difference between PIL samples, which suggests that no electrostatic interaction is involved once the counter surface does not carry any surface charge.

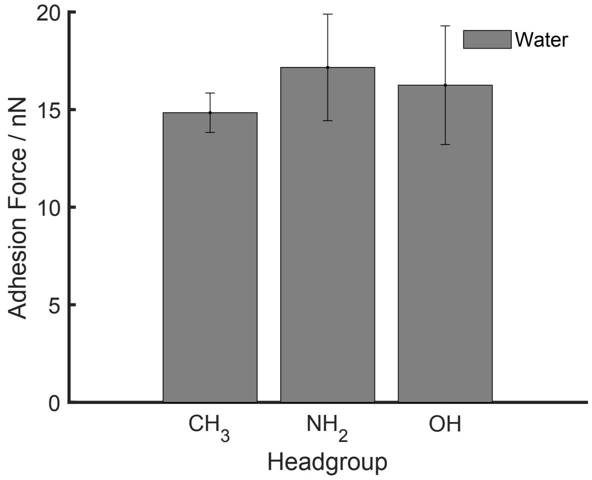

Figure S3. Surface adhesion acquired on the PIL samples as a function of different chemical groups, using colloidal force spectroscopy with a polystyrene particle (20 μm diameter).

References

1. Li, N.; Qu, R.; Han, X.; Lin, W.; Zhang, H.; Zhang, Z. J., The Counterion Effect of Imidazolium-Type Poly(ionic liquid) Brushes on Carbon Dioxide Adsorption. *ChemPlusChem* **2019,** *84* (3), 281-288.
2. Sednaoui, T.; Vezzoli, E.; Dzidek, B.; Lemaire-Semail, B.; Chappaz, C.; Adams, M., Friction Reduction through Ultrasonic Vibration Part 2: Experimental Evaluation of Intermittent Contact and Squeeze Film Levitation. *IEEE Trans. Haptic* **2017,** *10* (2), 208-216.
3. Green, N. C.; Bowen, J.; Hukins, D. W. L.; Shepherd, D. E. T., Assessment of non-contacting optical methods to measure wear and surface roughness in ceramic total disc replacements. *Proc. Inst. Mech. Eng., Part H: J. Eng. Med.* **2015,** *229* (3), 245-254.
4. Kaklamani, G.; Cheneler, D.; Grover, L. M.; Adams, M. J.; Anastasiadis, S. H.; Bowen, J., Anisotropic dehydration of hydrogel surfaces. *Prog. Biomater.* **2017,** *6* (4), 157-164.
5. Hejda, F.; Kousal, J. In *Surface Free Energy Determination by Contact Angle Measurements - A Comparison of Various Approaches*, WDS'10 Proceedings of Contributed Papers Part III, Charles University, Prague, Czech Republic, 2010; pp 25-30.
6. Kelley, T. W.; Schorr, P. A.; Johnson, K. D.; Tirrell, M.; Frisbie, C. D., Direct Force Measurements at Polymer Brush Surfaces by Atomic Force Microscopy. *Macromolecules* **1998,** *31* (13), 4297-4300.
7. Yamamoto, S.; Ejaz, M.; Tsujii, Y.; Matsumoto, M.; Fukuda, T., Surface Interaction Forces of Well-Defined, High-Density Polymer Brushes Studied by Atomic Force Microscopy. 1. Effect of Chain Length. *Macromolecules* **2000,** *33* (15), 5602-5607.
8. McLean, S. C.; Lioe, H.; Meagher, L.; Craig, V. S. J.; Gee, M. L., Atomic Force Microscopy Study of the Interaction between Adsorbed Poly(ethylene oxide) Layers: Effects of Surface Modification and Approach Velocity. *Langmuir* **2005,** *21* (6), 2199-2208.

9. Zhang, Z. J.; Edmondson, S.; Mears, M.; Madsen, J.; Armes, S. P.; Leggett, G. J.; Geoghegan, M., Blob Size Controls Diffusion of Free Polymer in a Chemically Identical Brush in Semidilute Solution. *Macromolecules* **2018,** *51* (16), 6312-6317.
10. Bielecki, R.; Benetti, E.; Kumar, D.; Spencer, N., Lubrication with Oil-Compatible Polymer Brushes. *Tribol. Lett.* **2012,** *45*, 477-487.
11. Bielecki, R. M.; Crobu, M.; Spencer, N. D., Polymer-Brush Lubrication in Oil: Sliding Beyond the Stribeck Curve. *Tribol. Lett.* **2013,** *49* (1), 263-272.
12. Sakata, H.; Kobayashi, M.; Otsuka, H.; Takahara, A., Tribological Properties of Poly(methyl methacrylate) Brushes Prepared by Surface-Initiated Atom Transfer Radical Polymerization. *Polym. J.* **2005,** *37* (10), 767-775.
13. Kilinic, E.; Hand, R. J., Mechanical Properties of Soda-Lime Glasses with Varying Alkaline Earth Contents. *Journal of Non-Crystalline Solids* **1998,** (429), 190 - 197.
14. Shackelford, J. F.; Han, Y.-H.; Kim, S.; Kwon, S.-H., *Materials Science and Engineering Handbook*. 4th ed.; CRC Press: Boca Raton, 2015.
